# Machine Learning to Quantitate Neutrophil NETosis

**DOI:** 10.1038/s41598-019-53202-5

**Published:** 2019-11-15

**Authors:** Laila Elsherif, Noah Sciaky, Carrington A. Metts, Md. Modasshir, Ioannis Rekleitis, Christine A. Burris, Joshua A. Walker, Nadeem Ramadan, Tina M. Leisner, Stephen P. Holly, Martis W. Cowles, Kenneth I. Ataga, Joshua N. Cooper, Leslie V. Parise

**Affiliations:** 1 Department of Biochemistry and Biophysics, University of North Carolina, Chapel Hill, NC 27599 USA; 2 Department of Pharmacology, University of North Carolina, Chapel Hill, NC 27599 USA; 3 Department of Computer Science and Engineering, College of Engineering and Computing, University of South Carolina, Columbia, SC 29208 USA; 4 Department of Mathematics, College of Arts and Sciences, University of South Carolina, Columbia, SC 29208 USA; 5 Department of Pharmaceutical Sciences, Campbell University, Buies Creek, NC 27506 USA; 6 EpiCypher, Inc. Durham, Durham, NC 27709 USA; 7 Department of Medicine, University of North Carolina, Chapel Hill, NC 27599 USA; 8 Lineberger Comprehensive Cancer Center, University of North Carolina, Chapel Hill, NC 27599 USA; 9 Present Address: Department of Medicine, University of Tennessee Health Science Center, 956 Court Avenue B330, Memphis, TN 38163-2116 USA; 10 Present Address: Department of Medicine, University of Tennessee Health Science Center, 956 Court Avenue D324, Memphis, TN 38163-2116 USA; 11 UNC Blood Research Center, University of North Carolina, Chapel Hill, NC 27599 USA

**Keywords:** Machine learning, Biological techniques, Assay systems, Cellular imaging, Neutrophils

## Abstract

We introduce machine learning (ML) to perform classification and quantitation of images of nuclei from human blood neutrophils. Here we assessed the use of convolutional neural networks (CNNs) using free, open source software to accurately quantitate neutrophil NETosis, a recently discovered process involved in multiple human diseases. CNNs achieved >94% in performance accuracy in differentiating NETotic from non-NETotic cells and vastly facilitated dose-response analysis and screening of the NETotic response in neutrophils from patients. Using only features learned from nuclear morphology, CNNs can distinguish between NETosis and necrosis and between distinct NETosis signaling pathways, making them a precise tool for NETosis detection. Furthermore, by using CNNs and tools to determine object dispersion, we uncovered differences in NETotic nuclei clustering between major NETosis pathways that is useful in understanding NETosis signaling events. Our study also shows that neutrophils from patients with sickle cell disease were unresponsive to one of two major NETosis pathways. Thus, we demonstrate the design, performance, and implementation of ML tools for rapid quantitative and qualitative cell analysis in basic science.

## Introduction

Machine learning (ML) is a branch of artificial intelligence built on the idea that computers can acquire knowledge through data and observations without explicit programming; they can then learn to generalize from examples and make predictions. ML has been used in analysis of genomics^1^, drug discovery^2,3^, modeling protein structures^4^ and disease diagnosis^5,6^. Few studies have used automated imaging technologies with ML in non-diagnostic and exploratory research-focused efforts such as cell quantification in animal models. Cell classification is laborious, as it relies on heavily supervised image analysis tools with the need for continuous user interaction from image pre-processing steps through feature extraction to final classification/labeling. Although the reduced error rates and exceptional learning speeds of ML have the potential to transform the field of cellular imaging, they have not been widely adopted in the biological sciences, in part because of a lack of testing and validation of such methods due to shortages of large datasets for training. Convolutional neural networks (CNNs) are deep learning algorithms commonly used in image recognition and classification. Their structure was inspired by the structure of the mammalian visual cortex, whose function is pattern recognition and computing complex object attributes. Here we demonstrate for the first time the feasibility of designing different CNNs to address key questions relating to the timely topic of neutrophil NETosis.

NETosis (cell death by Neutrophil Extracellular Traps) is characterized by chromatin decondensation, nuclear swelling and rupture, followed by DNA release in a net-like structure^7^. It constitutes a response to danger signals and is a vital part of the immune system evolutionarily conserved across multiple organisms^8–11^; however, excessive NETosis is associated with severe inflammation and disease progression, making it an important indicator, endpoint, and target in several clinical trials^12–19^.

To date, at least two distinct *in vitro* NETosis mechanisms have been identified: one is mediated by the enzyme peptidyl arginine deiminase (PAD4) and another by reactive oxygen species (ROS) generated following protein kinase C (PKC) activation and the granular protease, neutrophil elastase (NE)^20,21^. To decondense chromatin, PAD4 citrullinates histone arginine residues, causing a decrease in electrostatic interaction between DNA and histones; whereas NE degrades histone proteins. The exact role of ROS in NETosis is unknown. These two NETosis pathways utilize different biochemical mediators, for example histone citrullination is a marker for the PAD4-mediated but not the ROS-mediated pathway^22,23^. Targeting PAD4 with pharmacological inhibitors is successful in inhibiting NETosis *in vitro*^24^ and in animal models^25–27^; however, no targeted therapies for the ROS-pathway exist^27–31^. DNase-1 has also been used to digest the nuclear backbone of NETs and prevent ischemia-reperfusion (I/R) injury^32^, lung injury due to cigarette smoke or mechanical ventilation^33,34^, and deep vein thrombosis^35^. However, using DNase to dismantle NETs risks enhancing bacterial^36^, fungal^37^ and viral pathogen^38^ virulence. A better strategy to inhibit NETosis would be to prevent chromatin decondensation, the defining characteristic of NETotic cells for both pathways. Chromatin decondensation underlies the change in nuclear shape from multilobulated to spherical/ovoid and is a specific marker for NETotic cells that is distinct from necrosis and apoptosis^39^. The earliest attempts to quantitate NETosis involved measuring changes in the size and shape of neutrophil nuclei using fully-supervised image processing methods which suffer from the weaknesses of rule-based modeling^40–43^. Rule-based modeling is the use of fixed, often highly complex, rules crafted by domain experts to make predictions or decisions – and, in contrast with statistical learning, is extremely labor-intensive, highly limited in its ability to generalize, and constrained by human cognitive biases and finite imagination^44^.

There are two general strategies to quantitate NETosis and NETs. One focuses on the quantitation of the final product of NETosis, the extruded NETs and associated proteins by targeting citrullinated histones using ELISA, assessing the activity of DNA-associated NE and, image analysis to estimate the fraction of an area covered by NETs (NET area)^45–48^. This strategy forms the basis of the two commercially available kits to quantitate NETosis. Coelho *et al*. were the first to introduce a high-throughput version of NET area analysis using supervised machine learning method^47^. A limitation of this strategy is the increased risk for false positives given that DNA release also occurs following other processes such as necrosis. Additionally, in the absence of a specific marker such as citrullinated histone antibodies, quantitation of extruded NETs fails to provide information regarding the NETosis pathway involved.

The other main strategy to quantitate NETosis capitalizes on the discernable change that occurs in nuclear shape (image analysis of fixed and adherent fluorescently-labeled neutrophil DNA^42,43^, flow cytometry^49^ and imaging flow cytometry^50^), which involves assessing the fraction of NETotic nuclei in a mixed cell population. With this second strategy, investigators have used both adherent and non-adherent neutrophils. Flow cytometry-based methods including imaging flow cytometry are high-throughput and automated but require non-adherent NETotic cells. There is increasing evidence showing that neutrophil adhesion is an essential component of NETosis and that certain inducers require neutrophil adhesion to a matrix before NETosis can occur^51,52^. Therefore, one potential drawback to flow cytometry-based detection is the underestimation of the amount of NETosing cells. The method presented here falls within the second strategy of the quantitation of the number of NETotic nuclei in a mixed cell population. The improvement we introduce is the use of convolutional neural networks to classify NETotic and non-NETotic adherent neutrophils; efficiently process thousands of cell images captured by an automated microscope system; facilitate graded dose-response analyses; predict which of the two major NETosis pathways is involved; and distinguish NETotic from necrotic cells.

## Results and Discussion

**Figure 1:**
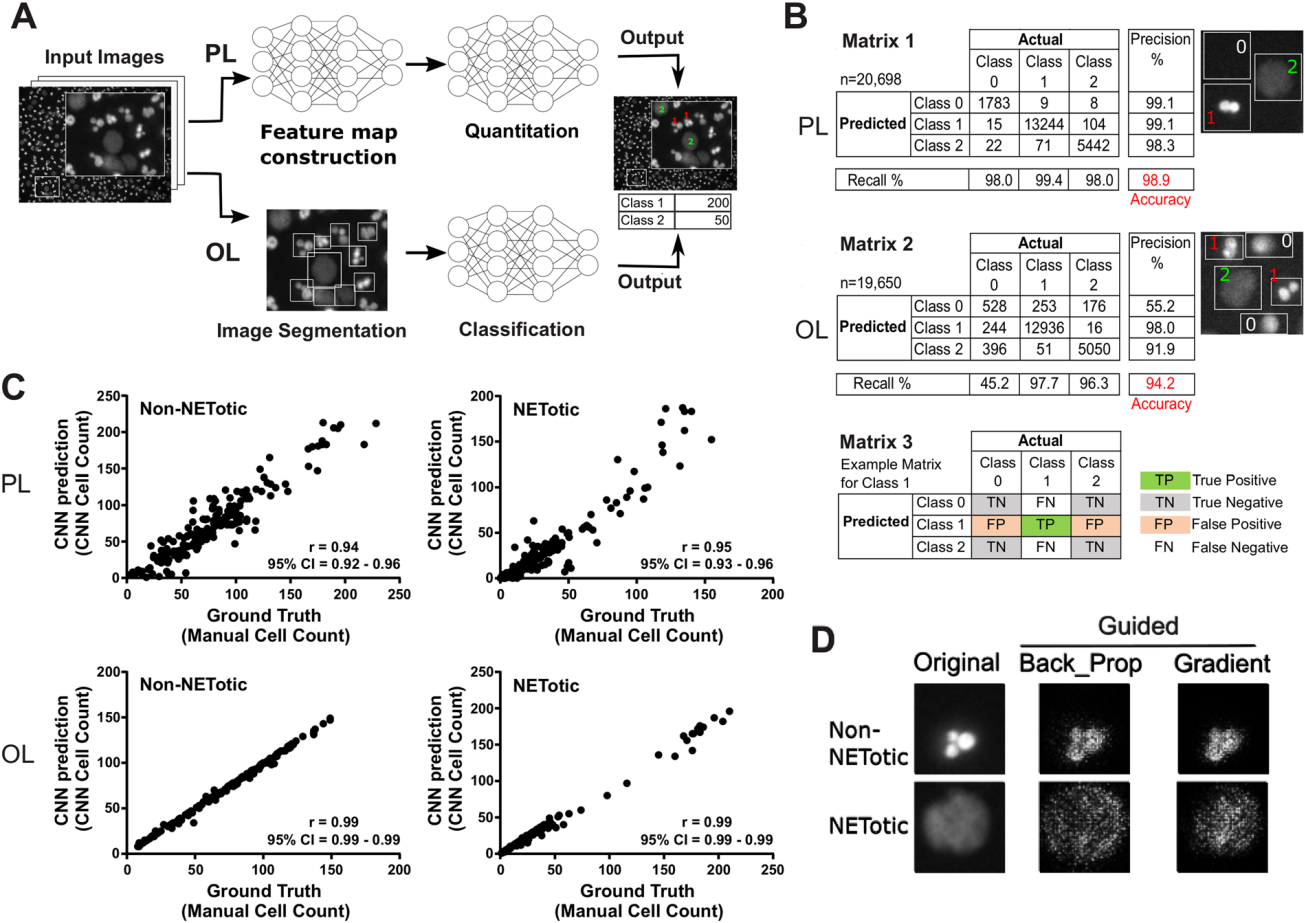
(**A**) The first panel represents one field (672 × 512 pixels of a 16-bit image) out of 36 fields in a typical image; the inset contains nuclei with the annotations 1 or 2 corresponding to non-NETotic and NETotic nuclei, respectively. The annotations were generated manually using ImageJ. The pixel-level (PL) classifier was trained by scanning the whole image (a total of 4032 × 3072 pixels) in 32 × 32-pixel patches and classifying each patch as a class 1 or 2 using the annotations found on the image. The object-level (OL) classifier uses drawn bounding boxes of variable dimensions around all objects identified in the image and uses the object in the bounding box as training data. (**B**) Confusion matrices were used to evaluate model performance on the holdout dataset excluding the training dataset. The n numbers represent the holdout dataset only. The numbers in red denote model accuracy, which is the percentage of total correct predictions by the CNN. Recall is the number of true positives divided by true positive + false negative or the fraction of actual true positive predictions identified correctly. Precision is the number of true positive values divided by true positive + false positive or the fraction of positive identifications that were actually correct. An example of what would constitute true positive, true negative, false positive and false negative for class 1 is shown in matrix 3. Matrices 1 and 2 represent confusion matrices for PL and OL respectively. The nuclei images adjacent to the matrices indicate which nuclei were labeled as class 0, 1 or 2 by the two different CNN. The major difference in training of the CNNs is the class 0 category. (**C**) Pearson’s correlation coefficient (r) was used to compare the quantification of PL and OL (CNN prediction) to that performed manually (ground truth). A total of 186 and 161 images containing hundreds of cells were quantified by PL and OL, respectively, with a confidence interval of 95%, p < 0.0001 for all obtained R values. Each dot represents an image that was counted manually and by a CNN for the total number of Non-NETotic (graphs on the left), and NETotic cells (graphs on the right) in an image. (**D**) Guided backpropagation as well as gradient-weighted class activation mapping were used to generate saliency maps evaluating the relative contributions of each pixel to the CNN’s prediction. The brighter a pixel appears on this map, the more salient it is in identifying the phenotype and the more value it has in determining the CNN’s prediction.

A large set of human-annotated neutrophil nuclei (103,874) were used to train and test two CNNs in identifying three classes of objects. Non-NETotic (class 1) nuclei are identified by the presence of lobules (2 or more) and bright fluorescent signal, whereas NETotic (class 2) nuclei are spherical and have a significantly diffuse fluorescent signal. The CNNs were trained by either pixel-level (PL), end-to-end or object-level (OL) classification techniques (Fig. 1A). The negative class for PL (class 0) was randomly selected pixels that did not have a signal (blank areas). For OL, class 0 denotes all objects not classified as 1 or 2, which may contain areas of spread NETs or nuclei not identifiable as one of the two classes. We have designed PL using an architecture with fewer hyper-parameters, enabling operations on commodity hardware without loss in classification accuracy. PL learns from annotated neutrophil nuclei without additional knowledge of the image or its objects. The first steps in PL training involves the building of a feature map or an activation map which is a representation of the input image (Supplementary Information, Fig. 1A). In contrast, OL requires high performance computing specialized hardware. Prior to training the OL network, all image objects were individually identified (Fig. 1A). The dataset was divided into a training set composed of 80% of the data, and a testing set composed of 20% of the data that served as out-of-sample (never “seen” by the network) examples for evaluation (Table 1). We assessed network accuracy, precision and recall using confusion matrices. We obtained accuracies of 98.9% and 94.2% respectively for PL and OL, signifying that if either CNN was given a new image of a nucleus, its prediction would be correct with the probabilities of 98.9% and 94.2% respectively (Fig. 1B, Matrices 1–2). Precision is a measure of how often the CNN is correct in predicting a positive event (true positive/true positive + false positive). Recall is an indicator of the sensitivity of the model and is calculated by taking the ratio of correct positive predications to the total actual positives (true positive/true positive + false negative). Given that our model is not binary, an example of what would constitute a true positive (TP), true negative (TN), false positive (FP) and false negative (FN) for class 1 is shown in Fig. 1B Matrix 3. PL achieved both high precision and recall values for classes 0–2. Although OL’s precision and recall were comparable to PL’s for classes 1 and 2, predicting class 0 was neither as precise nor as sensitive leading to the overall lower accuracy of OL. This slightly lower accuracy score is due to the more demanding discrimination task this model is trained to perform; class 0 for OL was a variegated combination of objects that are not labeled as class 1 or class 2. PL and OL quantitation accuracy were compared to manually annotated images containing several hundred nuclei per image by plotting CNN prediction versus manual cell count and performing a correlation analysis (Pearson’s r ≥ 0.94, p < 0.0001 for all obtained r values) (Fig. 1C). Correlation analysis was also performed on quantitation of 44 images using ImageJ. The performance of ImageJ in the quantitation of NETotic nuclei was better than non-NETotic (Pearson’s r = 0.74, 95% CI = 0.57–0.85 for NETotic and r = 0.42, 95% CI = 0.14–0.64 for non-NETotic, p < 0.01 for both values) as can be seen in Supplementary Information Fig. 2C. CNNs achieved much greater accuracy in quantitation of NETotic and non-NETotic nuclei as was expected.

**Table 1:** Breakdown of the number of nuclei used for training, validation and testing the PL and OL CNNs.

	Total No. of Nuclei used for:		
	Training	Validation	Testing
PL	83099	—	20774
OL	65323	17209	19650

Understanding how and why CNNs achieve such high performances is an active area of research involving approaches such as network output analysis and saliency maps. To investigate what the CNNs were learning that enabled accurate predictions, we obtained examples of image objects with low and high network output activation to gain insight into the models’ understanding of the data (Supplementary Information, Fig. 1B). Objects with high network activation are those with high probability of belonging to their assigned class and are more representative of that class. Although useful in visualizing a prototype of each class; this method is not informative as it fails to describe the most identifying feature(s) captured by the network of each class. The saliency method attempts to identify salient regions in a visual object by assigning a feature importance to each pixel. We obtained class saliency maps by comparing the gradient of output class in relation to an input image, essentially determining which pixels/regions in the input image are most responsible for the networks’ decision^53^. Saliency maps are analogous to heat maps and are a mechanism to visualize what the network is learning. On the saliency map, brighter pixels are more critical for object recognition/classification. We used two techniques to generate saliency maps, using 10 representative nuclei of each class (NETotic and non-NETotic) (Fig. 1D and Supplementary Information, Fig. 1C for additional examples of each class). The saliency map of class 1 shows two or more closely clustered pixel groups that strongly resemble the original input image, whereas the class 2 map shows a more dispersed pixel distribution with slight emphasis on one relatively central region in the nucleus. This region could represent the innermost core of the chromatin that has not been completely decondensed.

**Figure 2:**
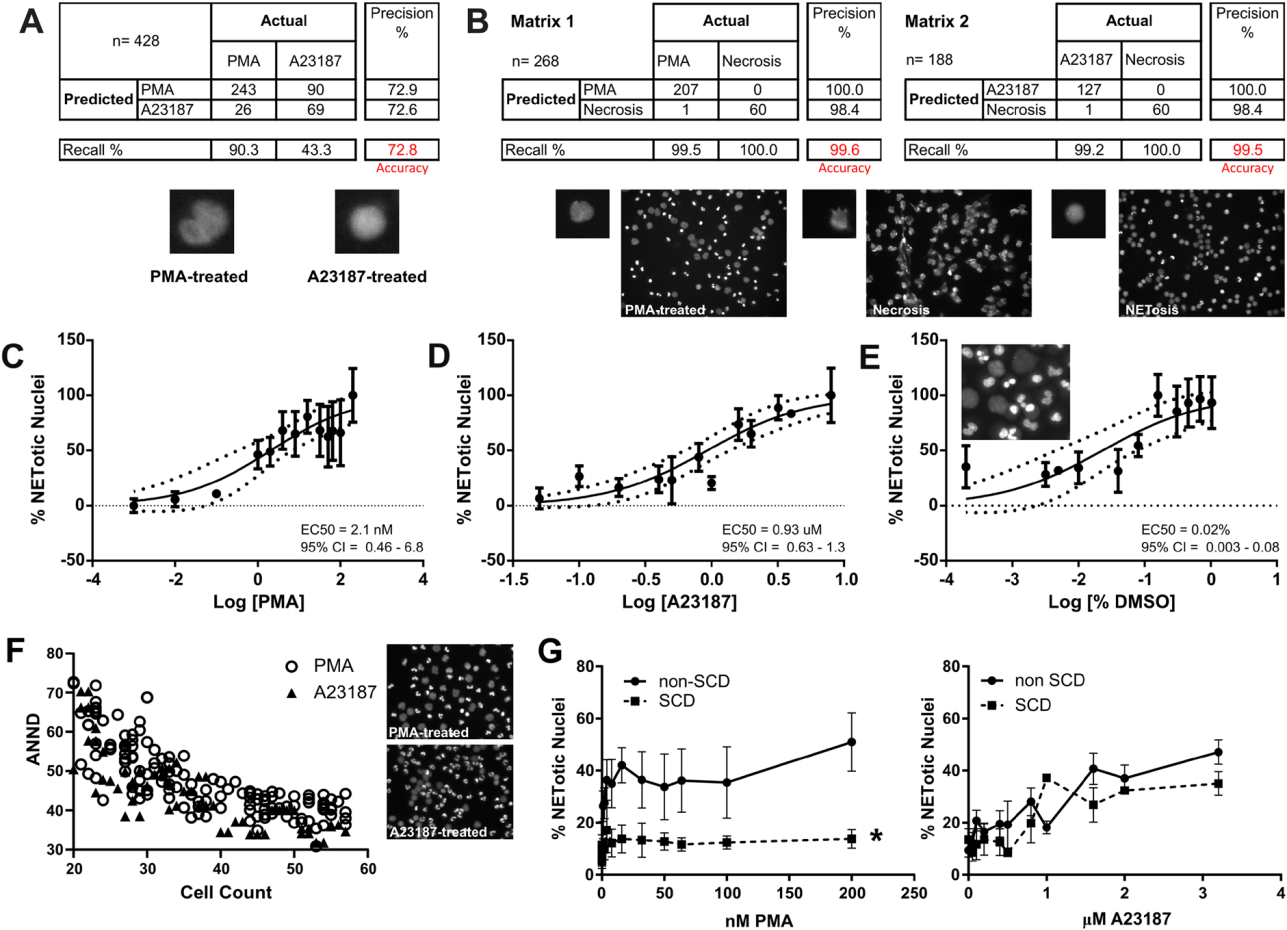
(**A**) A CNN trained on a total of 1286 individual NETotic nuclei (807 and 479 nuclei produced as a result of PMA or A23187 treatment, respectively). Treatment time was 120 min and we analyzed images where agonist concentration would result in 50% NETotic nuclei. Nuclei were cropped out of images in a bounding box of a fixed dimension and used for CNN training. The confusion matrix demonstrates CNN’s ability to differentiate between the two nuclei with an accuracy of ~73%. The dataset used for testing (n = 428) represents 25% of the total number of images and is a subset that was excluded from the training set. An example of a PMA- and A23187-treated nucleus is shown below the matrix. (**B**) Necrosis was induced by freezing neutrophils at −80 °C for 80 min on tissue culture plates. Comparison between necrotic and NETotic nuclei was performed similarly to that between PMA- and A23187-induced NETotic nuclei. A total of 244 necrotic, 506 A23187-induced and 833 PMA-induced NETotic nuclei were used for training the CNN. Although the number of necrotic nuclei used for training was relatively small, the CNNs achieved exceptionally high performance accuracies in differentiating between necrotic and NETotic nuclei as is seen in matrix 1 for PMA-treatment versus necrosis and matrix 2 for A23187-treatment versus necrosis. Images below the confusion matrices demonstrate the clear difference in appearance of NETotic and necrotic nuclei. (**C**) PMA was used to induce ROS-dependent NETosis and EC_50_ values were calculated as described in Methods. The percentage of DMSO in the highest agonist concentration was used as the vehicle control. EC_50_ was determined to be 2.1 nM for PMA and the 95% CI interval = 0.5–6.8, (n = 11). The 95% confidence bands are the dashed lines on the plot. (**D**) A23187 was used to induce PAD4-dependent NETosis and EC_50_ was determined to be 930 nM for A23187 with a 95% CI interval of 0.6–1.3, (n = 8). The percentage of DMSO in the highest agonist concentration was used as the vehicle control. The 95% confidence bands are the dashed lines on the plot. (**E**) DMSO is a widely used solvent for many NETosis agonists and inhibitors and we show that it acts as a NETosis agonist, reaching a maximum response of 30% at 0.16% DMSO (n = 3–5 data points for each concentration used), 95% CI for EC_50_ = 0.003–0.085. The dash lines represent 95% confidence bands. (**F**) Dispersion and clustering of NETotic nuclei differs depending on the type of treatment as can be seen in the images (right panels). CNNs were used to calculate and compare the clustering characteristics using Average Nearest Neighbor Distances which reveal significant differences (p ≤ 0.025) between PMA and A23187 treatments. Images chosen for analysis were those containing similar numbers of NETotic nuclei. The two sets of ANND values were compared using Kolmogorov-Smirnov test, which was run 10 times to obtain the following p-values: 0.0000022, 0.018, 0.0050, 0.00082, 0.0048, 0.000046, 0.025, 0.000077, 0.016, 0.0063. The average p-value is 0.0076. (**G**) The same cell isolation and agonist conditions were used to treat neutrophils from patients with SCD at steady-state (squares, dashed lines, n = 7) and non-SCD (circles, solid line, n = 11). The number of nuclei analyzed following PMA treatment is 752,824 and 410,238 for non-SCD and SCD groups respectively. Following A23187 treatment the number of nuclei analyzed was 314,037 and 94,759 for non-SCD and SCD groups respectively. Neutrophils from patients with SCD responded poorly to PMA treatment (two-way ANOVA, p < 0.05) suggesting impairment in the ROS-dependent NETotic pathway (graph on the left); in contrast no significant difference was observed between SCD and non-SCD donors in response to A23187 treatment (graph on the right), suggesting that PAD4-mediated NETosis is unimpaired in SCD.

As mentioned above, two distinct pathways of NETosis have been well identified, PAD4 and ROS, which can be activated by A23187 (a Ca^2+^-ionophore that activates PAD4), and PMA (a PKC activator that mediates ROS formation) respectively. Histone citrullination is unique to NETosis agonists that can activate PAD4 by causing calcium release (Supplementary Information, Fig. 2A)^54^. We hypothesized that in addition to the differences in their triggering and propagation mechanisms, the two pathways also might yield NETotic nuclei with different phenotypes that could be differentiated and quantified simultaneously by neural networks. This would forgo the need for specific staining for citrullinated histones (PAD4 pathway) or the colocalization of chromatin and neutrophil elastase staining (ROS pathway). We designed a CNN classifier to achieve this goal. The CNN was able to differentiate with 73% accuracy NETotic nuclei induced by either A23187 or PMA, even though nuclei appeared to be indistinguishable by traditional human microscopic image analysis (Fig. 2A, confusion matrix and images below).

We then addressed another important distinction required for accurate classification of neutrophil cell death, that of NETotic versus necrotic nuclei. Necrosis is generally identified as loss in cell membrane integrity and cell/nuclear swelling due to the influx of water and ions. Although specific information regarding chromatin changes during neutrophil necrosis are not available, it appears that chromatin condensation (commonly referred to as clumping) is a common occurrence in both apoptosis and necrosis^55^. Clumping does not occur during NETosis but the final stages of both NETosis and necrosis are similar in that chromatin is released extracellularly. We have chosen to differentiate NETosis and necrosis based on nuclear morphology by treating neutrophils with two different stimuli for necrosis, hypotonic shock and freezing at −80 °C^56,57^. Although the change in chromatin with hypotonic shock (example image in Supplementary Information, Fig. 1D) was easily distinguishable morphologically from that occurring during NETosis, the changes during freezing were less drastic and more like NETosis. Therefore, CNNs were trained to classify NETotic nuclei induced by A23187 or PMA and necrotic nuclei induced by freezing (Fig. 2B, Matrices 1–2). Remarkably, the two CNNs differentiated between NETotic and necrotic nuclei with >99% accuracy. Unlike the subtle differences between PMA- and A23187-induced NETotic nuclei, necrosis results in nuclei that are easily distinguishable from NETotic nuclei. Because CNNs can discriminate between PAD4- versus ROS-dependent NETosis and NETosis versus necrosis, they can potentially predict the signaling mechanisms associated with novel chemicals or disease states and facilitate the identification of appropriate inhibitors.

Having established highly consistent and accurate CNN performance, we implemented it to measure the concentration-response relationship of commonly used NETosis agonists in neutrophils isolated from healthy donors. We determined the EC50 values (concentrations resulting in 50% of NETotic nuclei) to be 2.1 nM for PMA and 900 nM for A23187(Fig. 2C,D). Moreover, we found that DMSO, the vehicle for both agonists induces NETosis at ≥0.08% v/v in culture media (Fig. 2E, graph and image), suggesting that treatment with agonists in solutions containing ≥0.08% DMSO should be avoided since it could result in an additive or synergistic effect on NETosis. The ability to quantitate EC50 values allows for a more precise assessment of new NETosis inhibitors or agonist potency in a high-throughput platform. In addition to using CNNs to perform straightforward object quantitation, we attempted to understand the spatial distribution of NETotic cells upon treatment with the two agonists, PMA and A23187. One impetus for this analysis was the observation that low PMA concentrations caused the appearance of adjacent NETotic and non-NETotic cells, suggesting the existence of distinct neutrophil populations with differing thresholds for PMA-induced NETosis (Fig. 2F, top image). Low A23187 concentrations did not induce this effect, but did induce NETotic neutrophil clustering, suggesting the possible release of a paracrine factor that potentiates NETosis in neighboring cells (Fig. 2F, bottom image) or activates neutrophil adhesion. To translate this important observation into a quantitative measurement, we capitalized on the ability of our ML model to locate cells by phenotype in microscope images and record their bounding boxes, to derive a list of the NETotic cells’ approximate geometric centers. These centers were then processed to compute the “average nearest neighbor distance” (ANND), a well-known metric for gauging degrees of clustering or dispersion (dating back to at least 1954, see Clark-Evans 1954)^58^. In general, if the ANNDs is small, the points (and therefore the cell centers) are more clustered whereas larger ANNDs indicate greater dispersion (Fig. 2F). Once the ANND statistics were computed for PMA- and A23187-treated NETotic cells, the two sets of numbers were compared using a general non-parametric, two-sample Kolmogorov-Smirnov^59^ test, to determine whether two sets of values were drawn from the same distribution. The average p-value was 0.0076 for the null hypothesis that the two cell sets were equally clustered/dispersed, with no results exceeding a p-value of 0.025 (Fig. 2F). From this result we conclude that using PMA or A23187 as a NETosis agonist results in NETotic nuclei with different clustering/dispersion patterns.

We are interested in the potential for CNNs to study NETosis in patient populations. We chose patients with sickle cell disease (SCD) given that chronic inflammation and hypercoagulability are well known complications in SCD^60,61^ and that NETs are thought to be important for both processes^13,62,63^. Previous studies report finding soluble components of DNA and nucleosomes in plasma from both SCD patients and humanized SCD mice^16^. Furthermore, plasma from SCD patients was found to cause NET production in neutrophils from healthy individuals^16^ leading to the conclusion that NETosis is associated with SCD pathophysiology. However, both studies use surrogate indicators for NETosis and did not measure NETosis directly in neutrophils isolated from SCD patients. Our technology allows us to quantitatively assess NETosis in neutrophils of patients with SCD at steady state (episodes where patients are not experiencing pain and other symptoms associated with crisis). SCD neutrophils responded similarly to neutrophils from non-SCD donors to A23187, indicating functional PAD4-dependent NETosis (Fig. 2G). In contrast, SCD neutrophils were insensitive to PMA (Fig. 2G), suggesting impairment in their ROS-dependent NETosis potential. Our results agree with previous studies reporting that neutrophils from SCD patients have reduced capacity for oxidative burst and therefore a reduction in ROS^64–68^. However there are also reports of increased oxidative burst and ROS generation in neutrophils in patients with SCD^69^. The decreased potential for NETosis in neutrophils from patients with SCD could explain their increased susceptibility to certain invasive bacterial infections as their neutrophils are unable to NETose and trap and kill bacteria efficiently. Our results could also reflect the response of neutrophils from patients with SCD to hydroxyurea or other pharmacological interventions. Additional studies with a larger patient cohort are certainly warranted and could elucidate important aspects in SCD disease mechanisms.

The method described herein is a major improvement over other available microscopy-based methods in NETosis research; it is far less laborious than manual object detection and classification, much more robust than semi-automated image analysis approaches, and substantially more accurate than the use of surrogate biomarkers. The simple (PL) and advanced (OL) classification models performed similarly well, suggesting that more complex CNNs are not needed for comparable classification tasks. In addition to straightforward quantitation of the percentage of NETotic nuclei in images, a task requiring substantial human effort and time, we demonstrate the ability of CNNs to perform tasks that would be very challenging if not impossible for the human eye. We demonstrate the application of CNNs in addressing both basic science and pre-clinical questions such as dose-response relationships of common NETosis inducers and the potential of patient neutrophils to NETose. Our analysis is especially useful in the quantitation of NETotic nuclei *in vitro* for graded dose-response relationships whereas analyses of the NET area, defined as the fraction of the regions in an image covered by NETs is more suitable for NET degradation and an all-or-none response^47^. A significant fraction of neutrophils have to extrude their DNA for any meaningful reading of NET area estimation which leads to decreased sensitivity of the method. Furthermore, the fact that extruded DNA can result from necrosis as well as NETosis, the measurements of extracellular DNA lack specificity for NET DNA. Specificity is vastly improved by using NET-specific markers such as an antibody targeting citrullinated histones; however, not all NETosis agonists cause histone citrullination^22,23^. Investigators have used traditional flow cytometry and imaging flow cytometry systems such as ImageStream to quantitate NETotic nuclei that are in suspension and not adhered onto a plate or a matrix^49,50,70^. There is a high risk of underestimating the percentage of NETotic neutrophils using imaging flow cytometry due to the reduced ability of non-adherent neutrophils to NETose after stimulation with certain agonists. It was shown that the bacterium *Acinetobacter baumannii* inhibits NETosis in human neutrophils by reducing neutrophil adhesion to glass slides^51^. Furthermore, it appears that some NETosis inducers such as LPS are dependent on neutrophil adhesion and substrate elasticity whereas others such as PMA are not^52^. Treating human neutrophils with a function blocking antibody to the integrin Mac-1 (αMβ_2_) results in reduced NET deployment; whereas mice lacking the β_2_ subunit of the integrin adhesion receptor have a reduced NETosis response to hantavirus infection^71,72^. Similarly blocking of the integrin LFA-1 (αLβ_2_) inhibits NETosis following endotoxin challenge^73^. DNA Area and NETosis Analysis (DANA) is an area-based analysis that is the most similar to the one proposed in the current work as it detects both NETotic nuclei and NET area^41^. DANA uses ImageJ and Java to automate image segmentation and nuclei quantitation. As discussed in the introduction, ImageJ analysis falls into the category of rule-based modeling and is highly dependent on operator-defined features and continuous operator supervision and adjustments.

Machine learning introduces much needed impartiality in image analysis by allowing the machine to determine all the features specific to NETotic and non-NETotic nuclei. By choosing to focus on the unique nuclear shape change that occurs in NETosis, we eliminate the need for a universal NETosis-specific marker, and we also facilitate analysis of graded pharmacological responses. To ensure the success of the current method, NETosing neutrophils should only be treated for the minimum time required to cause nuclear shape change. This requirement is easily met given that on average the NETosis process takes 4 hrs to reach completion (extrusion of NETs) *in vitro*. Longer treatment times or very potent stimuli may lead to the release of DNA before nuclear shape change is captured. Recent findings reveal that many scientific discoveries made using machine learning have been difficult to reproduce due to the limited volume of data used for machine learning training. We believe that our models performed exceptionally well due to the large volume of annotated data used for training the CNNs^74^. When such data volumes are not attainable, data augmentation techniques such as subtle image rotations can be employed^75^.

## Methods

### Sample collection

All subjects gave written informed consent in accordance with the Declaration of Helsinki. Consent was obtained by trained research personnel. The study was approved by the Institutional Review Board at the University of North Carolina at Chapel Hill (UNC) under IRB # 17-3148. Patients with SCD at steady state were recruited from the SCD clinic at UNC. Blood samples were collected via venipuncture from healthy volunteers in the research laboratory of LVP by trained personnel/phlebotomist or donated by the UNC Healthcare Blood Donation Center located at the NC Cancer Hospital. Approximately 5 mL of blood was collected in vacutainers coated with EDTA (BD 367863). Inclusion and exclusion criteria for both SCD and non-SCD volunteers are outlined below.

#### SCD

Inclusion:

- Diagnosis of SCD (genotype SS, SC or S/β^0^ thalassemia).
- Non-crisis at time of enrollment and no acute pain episodes in the previous 4 weeks.

Exclusion:

- Inability to provide informed consent based on the judgment of study personnel. <18 years of age.
- Anticoagulation therapy
- Recent history of hemoglobin levels of <6 g/dL
- Less than 3 months have transpired since last transfusion.
- Pregnancy

#### Non-SCD

Inclusion:

- >18 years of age.
- Healthy and of either sex

Exclusion:

- Inability to provide informed consent based on the judgment of study personnel. <18 years of age.
- Anticoagulation therapy
- Taking Tylenol and/or Advil within 3–4 days prior to blood draw
- Pregnancy

### Neutrophil isolation and treatment

Following collection, the blood sample was handled under sterile conditions throughout treatment and up to cell fixation to prevent activation during the isolation process. Neutrophil isolation was performed using EasySep Direct Human Neutrophil Isolation Kit (STEMCELL Technologies, British Columbia, Canada, 19666) based on negative selection from whole blood. In brief, unwanted cells are cross-linked to magnetic particles via a tetrameric antibody complex. The isolation procedure is rapid (approximately 30 min). An average of 4.4 × 10^7^ and 5.5 × 10^7^ total neutrophils can be isolated from 5 mL of blood from non-SCD and SCD donors, respectively. Neutrophil isolation from SCD patients by density gradient centrifugation is challenging due to the altered rheology of SCD blood and the ineffectiveness of RBC sedimentation by dextran due to the low RBC aggregation index and rate in SCD patients.

Neutrophils were plated at a density of 3 × 10^6^/well in 96-well plates (Greiner Bio-One, Germany, 655986). This seeding density is optimal for allowing adequate spacing between nuclei once chromatin decondensation takes place and facilitates object identification and image analysis by CNNs. Cells were suspended in RPMI 1640 L-glutamine media (Gibco/Thermo Fisher Scientific, New York, USA, 11875-093) supplemented with 0.5% FBS (Gibco/Thermo Fisher Scientific, Grand Island, USA, 10438018) and incubated at 37 °C, 5% CO_2_. Cells would adhere during the first hr followed by treatment with vehicle or agonist for an additional 2 hrs; the total time neutrophils spend in culture is 3 hrs. Each assay condition was run in duplicate and for each well treated with a designated concentration of agonist, a neighboring well was treated with vehicle only. This design allowed for the determination of the effect of vehicle alone on NETosis. Cells were treated with lower than or equal to the maximum percentage of DMSO the did not cause significant NETosis. Necrosis was induced using freezing at −80 °C or hypotonic shock. Following agonist incubation, the supernatant was removed, and neutrophils were fixed with 4% paraformaldehyde for 20 min before staining.

### Cell staining and imaging

Following fixation, nuclei were stained using 0.05 μM of the nucleic acid stain SYTOX Green (Invitrogen/Molecular Probes, Oregon, USA, S7020). The BD Pathway Bioimaging 855 system was used to obtain 36 total adjacent images/well. Acquired images were stitched together by BD AttoVision 1.6 acquisition software to yield one image of 4032 × 3072-pixel dimensions that represents approximately 16% of the total surface area of the well in a 96-well plate. Staining for citrullinated histone H3, myeloperoxidase (MPO) and neutrophil elastase (NE) were performed using ab5103, ab9535, and ab68672, respectively (Abcam, Cambridge, MA, USA). A goat anti rabbit Alexa 594 secondary antibody was used for visualization. Image acquisition for double staining was performed using an Olympus IX81 inverted wide field microscope.

### Cell annotation

A total of 103,874 individual nuclei were manually annotated using ImageJ by 4 different investigators blinded to the treatment groups. Approximately 80% of this annotated dataset was used to train the developed CNN classifiers. Annotated neutrophil nuclei were designated as type 1 or non-NETotic if clear normal nuclear segmentation is present whereas type 2 or NETotic nuclei were labeled based on lack of clear nuclear segmentation and diffuse SYTOX Green stain. Nuclei that could not be identified as belonging to either of the two classes or spread NETs were labeled with either 4 or 5, respectively. The CNN classifiers were not trained and did not learn to identify classes 4 and 5 as we opted to train the classifiers on objects that could easily be differentiated.

### ImageJ analysis

Image processing of wide-field image sets was accomplished using automated scripts using a freely available software platform ImageJ (version 1.52o). For ImageJ, each FITC image in a directory was opened sequentially, background subtraction applied to correct uneven illumination, and primary objects were defined using Isodata segmentation. Small objects (having areas under 100 pixels) and cells touching the image periphery were removed from data set. The resulting objects were divided into two groups by defining large diffuse cells having a solidity value (Area/Convex Area) of above 5.677 and smaller cells below that threshold (Supplementary Information, Fig. 2C).

### PL CNN analysis

Note: A glossary of key terms used in describing the CNN methodology is included in Supplementary Information.

The Pixel-Level (PL) convolutional neural network (CNN) analysis proceeded in two stages.

#### Stage 1. Cell recognition/discrimination training

Data Preprocessing: First, patches of each 512 × 672 image indicating NETotic, non-NETotic, or negative class (neither phenotype) were extracted by centering a 32 × 32 box at each annotation mark (for the positive classes) and a random 32 × 32 box not containing an annotation mark (for the negative class). The result was 28865 negative class images and 75009 positive class images, for a total of 103874 images, which were then split evenly into 80% training and 20% testing (hold-out) data.

Network Description: The proposed network used an architecture inspired by VGGNet^76^ although substantially shallower to allow for training on commodity laptop hardware and to limit model complexity. There were four convolutional layers (64 filters, each 3 × 3), with 2 × 2 max-pooling and 25% dropout used between the second and third layers. The last convolutional layer was followed by a 256-node fully connected layer, and finally by a 3-node fully connected layer to indicate the three class probabilities. All activation functions were rectified linear (“ReLU”) except for the last layer, which was softmax. Weights were initialized randomly before training.

Training: The training dataset did not use augmentation, and categorical cross-entropy was used as the loss function. The network was trained with a learning rate of 0.001, a decay of 1e-7, and a momentum of 0.9.

#### Stage 2. Training for cell quantitation

Data Preprocessing: The original, unannotated images (training and testing) were then processed by applying the network trained in Stage 1 to every 32 × 32 patch of the image, which generated 480 × 640 × 3 feature maps: 740 for training and 186 for testing. The generated feature maps were associated with counts by phenotype from the original annotations.

Network Description: The proposed network again used an architecture inspired by VGGNet, but even shallower than in Stage 1 in order to limit model complexity because of the small training set size. There were two convolutional layers (32 filters, each 3 × 3), with 2 × 2 average-pooling used between them and 2 × 2 max-pooling used after them. This was followed by a 256-node fully connected layer, and finally by a 2-node fully connected layer to indicate the two object counts. All activation functions were rectified linear (“ReLU”).

Training: Mean absolute error was used as the loss function. The network was trained with a learning rate of 0.001, a decay of 1e-7, and a momentum of 0.9. The network was trained for 30 epochs.

Overall testing: The 186 feature maps generated from test data in Stage 2 were processed by the cell quantitation CNN generated in Stage 2, and the CNN-generated counts (estimated counts) were compared to the manually annotated images (ground-truth).

### OL CNN analysis

The pipeline consists of preprocessing both annotations and images and training the proposed network to classify the extracted patches of the cells.

#### Dataset preprocessing

Repeated erosion and dilation operations remove existing noise and clarify the boundary between the cells. Afterwards, all the connected regions and their centers are detected. At this point, a 112 × 112 patch is utilized, and the patch was labeled according to the annotation point closest to the center of the patch. The entire dataset was split into 65:15:20 ratio to create training, validation and testing datasets respectively. As part of image preprocessing, all images were normalized by subtracting the mean of image matrix from image matrix and dividing the result by the standard deviation of image matrix.

#### Network description

The proposed network was inspired by DenseNet^77^, in particular, the 121-layer configuration. The last layer of the DenseNet-121 was replaced by a fully connected layer with 3 hidden units in order to output 3 classes (Class 0, 1 and 2) of nuclei. All layers except the last fully connected layer were initialized with weights pre-trained on ImageNet and the last layer was randomly initialized.

#### Training

The training dataset was augmented by applying random rotations between −10 degrees to +10 degrees and horizontal/vertical flips in addition to original patches. Finally, each image was resized to 112 × 112 pixels, which was the input of the designed CNN training network. The network was trained with a learning rate of 0.001 and momentum of 0.9. The learning rate was reduced by 1e-5 for every 10 iterations. The network was trained until it starts to over fit, *i.e*., the validation loss starts increasing when the training loss is decreasing.

#### Testing

For testing, images not used in training and never seen by the CNN were used. The images were only normalized and fed to the network. No data augmentation was applied in the testing phase.

Table 1 summarizes the breakdown of the number of data points used for training/validation and testing of each model.

### Phenotype discrimination convolutional neural network (CNN)

#### Data preprocessing

First, 100 × 100 patches of each image indicating the location of a cell were extracted by manually cutting out a box around each patch. A total of 1,076 PMA-treated cell images and 638 A23187-treated cell images were each split evenly into 75%/25% training and testing (hold-out) data.

#### Network description

The proposed network used an architecture inspired by VGGNet^76^, although substantially shallower to allow for training on commodity laptop hardware and to limit model complexity. There were nine convolutional layers (64 filters, each 3 × 3), with 2 × 2 max-pooling and 25% dropout used after each three layers. The last convolutional layer was followed by a 256-node fully connected layer, and finally by a 2-node fully connected layer to indicate the two class probabilities. All activation functions were rectified linear (“ReLU”) except for the last layer, which was softmax. Weights were initialized randomly before training.

#### Training

The training dataset used no image augmentation, and categorical cross-entropy was used as the loss function. The network was trained with a learning rate of 0.001, a decay of 1e-7, and a momentum of 0.9. The network was trained for 100 epochs.

#### Testing

The 428 test images were processed by the trained CNN, and the class probabilities were set at a threshold of 50% to construct a confusion matrix.

We have posted the source code at https://github.com/Modasshir/cell-classification-journal. The source code contains training and testing code along with instructions. The trained weights for the entire dataset can also be downloaded to verify the accuracy on a sample dataset included in the project on the GitHub. The source code has been validated by an independent investigator.

### Saliency maps

We employed techniques described previously to generate saliency maps, a method for visualizing the internal “thought process” of a CNN^78^. Saliency maps assign to each pixel of an input image a value indicating the amount of influence on the final classification decision that pixel had; thus, highlighting regions of the image that were important in the decision-making process.

We used two methods: “Guided Backpropagation Class Activation Mapping” (“Guided Backprop”) and “Guided Gradient-weighted Class Activation Mapping” (“Guided Grad-CAM”) to calculate saliency maps. The former method identifies pixels which were highly influential for the final classification probability by tracing derivatives of node activations backwards through the network from the positive class node while discarding negative influences (*i.e*., derivatives). The latter method adds additional information to this process by also measuring the extent to which pixels occurred in an image region with a cumulatively positive effect on the final classification probability. Guided Grad-Cam is one extra step after Guided Backprop. After backpropagation, we receive the contribution of each image pixel both positive and negative. However, in the grad-cam step, negative contributing pixels are removed.

### Analysis of dose-response data

Datapoints were plotted as log [agonist concentration] versus response (% NETotic nuclei). All data were normalized to a starting response of 0 and a plateau of 100%, which were defined by the smallest and the largest means of the dataset, respectively. The 95% confidence bands are represented by the dashed lines and the 95% confidence interval are displayed on the plot. The range of concentrations used for DMSO were 0.0002% to 1.04% in media, (n = 3–5); PMA was used at 0.001–200 nM, (n = 11); and A23187 was used at 0.05–8 µM, (n = 8). The percentage of DMSO found in the highest agonist concentration was used as the vehicle control for PMA and A23187 groups. Comparison between SCD (n = 7) and non-SCD (n = 11) dose-response curves was performed using 2-way ANOVA using the mixed-effects model due to the presence of missing datapoints. The total number of nuclei analyzed for the non-SCD control group is 1,066,861 and for the SCD group is 504,997. Interactions between dose and genotype were deemed significant if p < 0.05. GraphPad Prism version 8.1 was used for generating graphs and statistical analysis.

### Computation of average nearest neighbor distance

Average nearest neighbor distance was calculated to quantitate agonist-induced neutrophil clustering. Because all images are the same size, no normalization is needed for this statistic, simply the average, across all points, of the ordinary Euclidean distance to their nearest neighbor. However, because the number of cells in an image will affect the ANND, the smaller of the two data sets (A23187) was resampled using the larger dataset (PMA) so that the cell-count marginals of the two-dimensional distribution of (cell-count, ANND) pairs were the same.

For ANND analysis, 24 PMA images and three A23187 images (4032 × 3072 resolution), were each cut into 6 × 7 rectangles for processing, resulting in 1008 A23187 images and 125 PMA images, since some images had no cells visible. These smaller images were then processed by the CNN to perform instance segmentation and extract the locations of the cell centers for each phenotype. Any images containing fewer than 20 NETotic cells were discarded to reduce noise, resulting in 709 A23187 images and 48 PMA images. We then computed the average nearest neighbor distances of NETotic cell centers in each image efficiently using the NearestNeighbors.fit() function from the Python package scikit-learn. At this point, we had a pair of numbers for each image: a cell count and an ANND. The pairs of PMA output numbers were culled so that it had approximately same n-size as the much smaller A23187 image set, uniformly at random with probability equal to the ratio of the image set sizes. The maximum of each set’s cell count was computed, the minimum of the two results was calculated, then all pairs (cell-count, ANND) from the two sets with cell-count larger than this max were removed. We resample the A23187 point set S in order to ensure its cell-count (empirical) marginal distribution matches that of the PMA point set. In particular, we create a new point set S’ from S by taking each point P from the PMA set, finding a point Q in S whose cell count is as close as possible to that of P (ties broken randomly), and then inserting Q into S.

### Declaration of interests

EpiCypher is a technology development company that does not intend to commercialize the present technology currently.

## Supplementary information

Supplementary Information

## Data Availability

The datasets generated during and/or analyzed as part of the current study are available from the corresponding author on reasonable request.
