# Supplementary material for: Machine Learning to Quantitate Neutrophil NETosis
Source: Sci Rep. 2019 Nov 15;9:16891. doi: 10.1038/s41598-019-53202-5 (PMC6858304; doi:10.1038/s41598-019-53202-5)
Supplement: Supplementary file 1 — Supplementary Information [file 41598_2019_53202_MOESM1_ESM.pdf]

Title: Machine Learning to Quantitate Neutrophil NETosis

Authors: Laila Elsherif [1*§], Noah Sciaky [2], Carrington A. Metts [1], Md. Modasshir [3], Ioannis Rekleitis [3], Christine A. Burris [4], Joshua A. Walker [1], Nadeem Ramadan [1], Tina M. Leisner [1], Stephen P. Holly [5], Martis W. Cowles [6], Kenneth I. Ataga [7#], Joshua N. Cooper [4&] and Leslie V. Parise [1,8]

Affiliations:

[1] Department of Biochemistry and Biophysics, University of North Carolina, Chapel Hill, NC.
[2] Department of Pharmacology, University of North Carolina, Chapel Hill, NC.
[3] Department of Computer Science and Engineering, College of Engineering and Computing, University of South Carolina, Columbia, SC.
[4] Department of Mathematics, College of Arts and Sciences, University of South Carolina, Columbia, SC.
[5] Department of Pharmaceutical Sciences, Campbell University, Buies Creek, NC.
[6] EpiCypher, Inc. Durham, NC.
[7] Department of Medicine, University of North Carolina, Chapel Hill, NC.
[8] Lineberger Comprehensive Cancer Center, University of North Carolina, Chapel Hill, NC.

Supplementary Figure 1

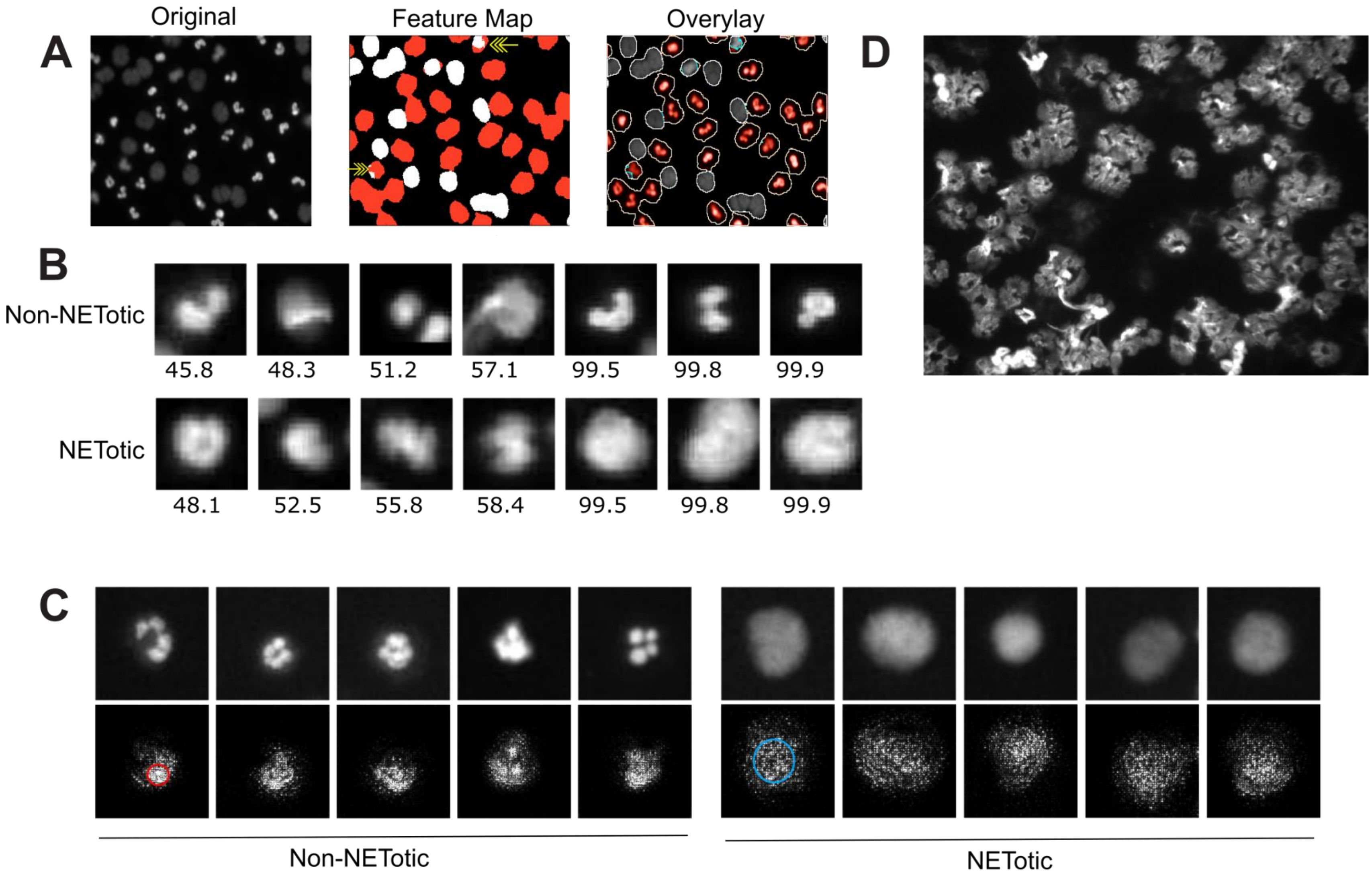

Supplementary Figure 1. A) For PL CNNs, a probability feature map (middle panel) identifies the coordinates of each class in an image and precedes any quantitation task. The feature map shows a probability for each of the 32 x 32 pixel windows analyzed in an image. Red and white overlay colors denote high probability that PL has determined that a particular pixel group is type 1 or 2 respectively. The yellow arrows highlight two examples of nuclei with regions classified as type 1 as well as type 2. PL's confusion in this example can be attributed to an edge effect and is the reason for our decision to disregard any nuclei positioned on the edges. The second yellow arrow highlights a nucleus that is presenting mostly as a type 1 for the exception of two small regions on the periphery which are labeled as a type 2. This effect could be due to the image of the nuclei being out of focus and therefore partially resembling a type 2. Those nuclei would be considered a type 1 since the majority of their feature map is indicating that the nucleus is a type 1. The third panel represents an overlay of the feature map on the original image highlighting a remarkable accuracy of the CNN in its predictions. B) The top and bottom rows of images represent non-NETotic and NETotic nuclei respectively, labeled with their CNN activation score. The higher the score the more likely that the CNN will assign that nucleus a type 1 or a type 2 classification given their close resemblance to a class prototype. C) Guided_Grad_Cam saliency maps of several non-NETotic and NETotic nuclei show one or more closely clustered pixels located at the periphery of the nucleus (red circle) that the network deemed essential to the identification of the class. For NETotic nuclei, a wider distribution of pixels in the center of the nucleus (blue circle) appear to be most essential to identification. D) Necrosis was induced by hypotonic shock using water treatment for 30 s followed by fixation and staining with sytox green.

Supplementary Figure 2

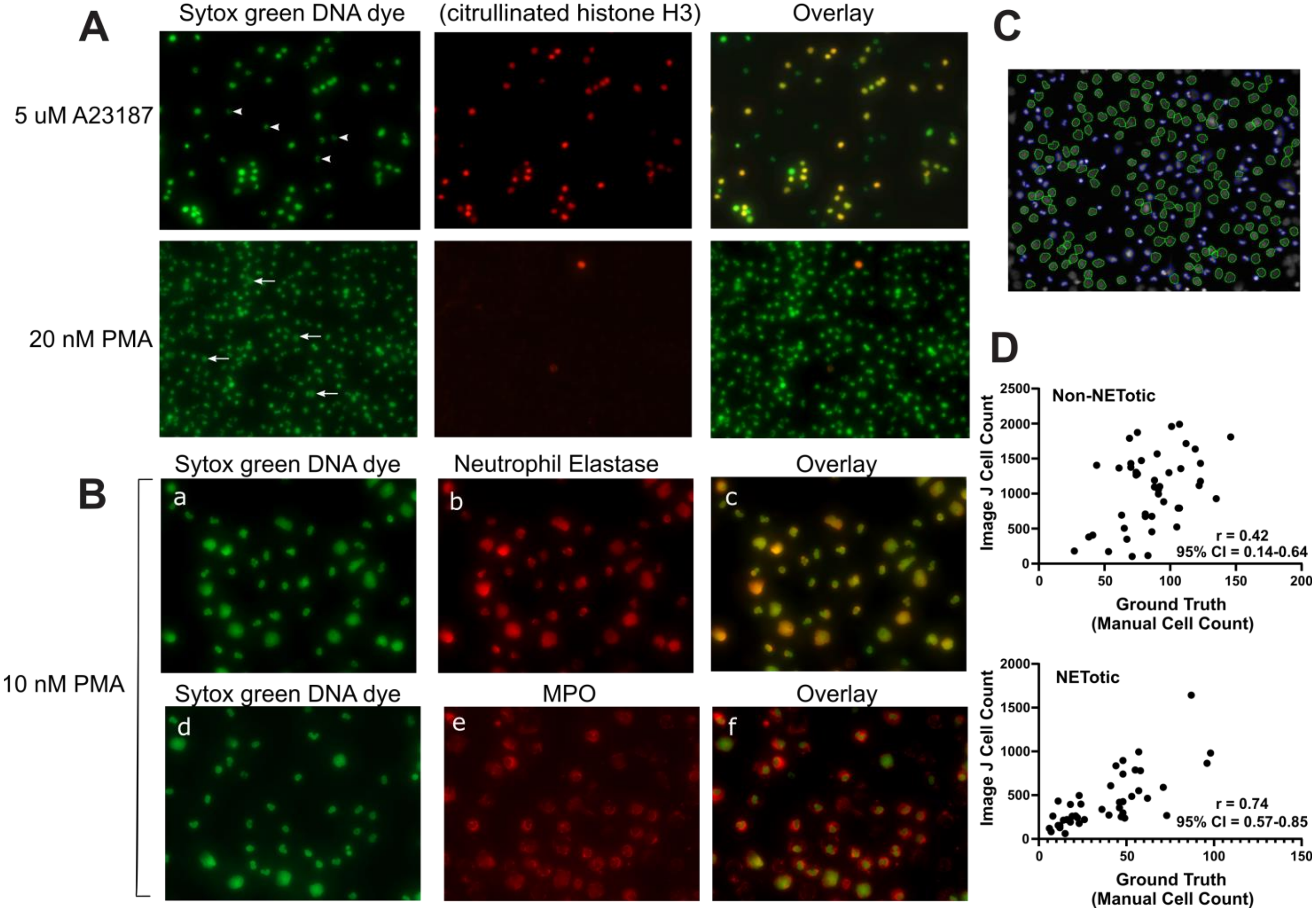

Supplementary Figure 2. A) Histone citrullination is a phenomenon specific to some but not all NETosis agonists. Neutrophils from non-SCD healthy donors were treated with either the Ca^{2+}-ionophore, A23187, or the PKC activator, PMA for a total of 3 hrs at the concentrations specified above. Cells were stained with either sytox green to visualize DNA, anti-histone H3 (citrulline R2 + R8 + R17 antibody, Abcam 5103) for citrullinated histone H3 or both. Images a-c correspond to neutrophils treated with A23187 and NETotic nuclei stain positive for citrullinated histone H3 whereas non-NETotic nuclei (white arrowheads) do not. Overlay image in c clearly shows colocalization of sytox green DNA dye and citrullinated histone H3. However, treatment of cells with PMA does not result in histone citrullination as most cells stain negative for citrullinated histone H3 despite the presence of decondensed nuclei indicating NETosis (white arrows in panel d) visualized with sytox green DNA dye. Image magnification is 20X. B) NETosis following PMA treatment results in the co-localization of neutrophil elastase (NE) and DNA but not myeloperoxidase (MPO). PMA treatment time and conditions are like those used for figure 2A above. Following treatment, neutrophils were stained with either NE or MPO antibodies to assess the co-localization of either signal with DNA. Notable overlap of NE and DNA can be observed with PMA treatment (image c) whereas MPO and DNA appear to be in different regions in NETotic neutrophils. Image magnification is 40X. C) An example image representing the segmentation we performed using ImageJ. Image objects were divided into two groups, large diffuse cells with a solidity value (Area/Convex Area) of above 5.677 (green colored objects) and smaller cells below that threshold (blue colored objects). Objects on the periphery of the image were excluded from the count. D) Pearson's correlation coefficient (r) was used to compare the quantification by ImageJ to that performed manually (ground truth). A total of 44 images were quantified by ImageJ. Confidence interval of 95 % was used and $p < 0.01$ for all obtained r values. Each dot represents an image that was counted manually and by ImageJ for the total number of Non-NETotic (top graph), and NETotic (bottom graph) cells.

Glossary

CNN classifier*: A "Convolutional Neural Network" which classifies input data by application of convolutional filters in addition to other modes of activation to perform feature extraction on geometric (often, image or video) data in an analogous manner to biological visual processing.*

Confusion matrix*: The matrix of values whose (C1, C2) entry, for classes C1 and C2, is the number of data points of true class C1 which were classified as C2 by an artificial neural network. Note that this includes the "correct" entries where C1=C2. Used to visualize the performance of a classification model.*

Convolutional Layers*: In an artificial neural network, a layer of artificial neurons which apply a linear function to their inputs via multiplication by an array of weights (a filter).*

Cross-entropy*: A measure of information loss (in bits) for assumption of an incorrect probability distribution.*

Decay*: Scheduled decreases in the learning rate of machine learning models.*

Deep learning*: A family of paradigms in machine learning which use many layers of feature extraction to build up semantically rich representations of data which can be processed into useful signals. Deeper models generally imply longer and more complex causal relationships between input and output.*

Dropout layer*: A layer of connections in a neural network of which random subsets are discarded temporarily during training. This is done to eliminate model over-fitting to a training dataset.*

Erosion and dilation operations*: Iterative removal of pixels surrounded by lower-valued pixels ("erosion", which reduces edge noise and approximately inverts dilation)*

and expansion of higher-valued pixel regions to neighboring pixels ("dilation", which smooths boundaries and approximately inverts erosion).

***Feature extraction**: The assignment of a value to data which measures some property thereof.*

***Feature/activation map**: A geometric representation of neuron activations – often the overall output of a neural network – used to visualize learning behavior and/or provide rich semantic features on a per-pixel basis for further (downstream) processing.*

***Filter / kernel**: An array of weights to be convolved with a correspondingly sized array of input values.*

***Fully connected layer**: A layer of connections in a neural network in which every upstream neuron is connected directly to every downstream neuron.*

***Ground truth**: The true value of a data point's class or value to be predicted by a model.*

***Hyper-parameters**: Architectural and algorithmic choices made in design of machine learning models (as opposed to parameters, which are values learned during training).*

***Image matrix**: The matrix representation of an image, with one value per pixel and channel (brightness, hue, saturation, redness, opacity, etc.).*

***Kolmogorov-Smirnov test**: A non-parametric statistical test to measure the likelihood that two samples were drawn from the same distribution, or that one sample was drawn from a reference distribution.*

***Learning rate**: The aggressiveness with which parameters/weights are adjusted to improve objective functions during learning.*

***Loss function / error function**: The penalty incurred during training for incorrect predictions. Machine learning algorithms learn by attempting to minimize the loss/error, often by gradient descent.*

***Machine learning**: Algorithms and software which learn from observations via modeling and inference in order to classify, make predictions from, or carry out tasks in response to data. Generally, machine learning is considered a subset of artificial intelligence.*

***Max-pooling**: A layer in neural networks that reduces the spatial dimension of its inputs by down-sampling using the maximum value of a region.*

***Momentum**: A parameter which controls how aggressively learning pursues a successful change in weights, designed to minimize oscillations during learning.*

***Negative class**: The subset of a training set for identification, classification, or segmentation tasks in which no feature (often, an object) of interest to the user is present.*

***Network accuracy**: The fraction of data points given to an artificial neural network trained on a classification task which are correctly classified.*

***Network output activation**: The value of an artificial neural network's output when processing a given input.*

***Network precision**: For each class C, the fraction of data points given to an artificial neural network trained on a classification task which were assigned class C, among all those whose true class is C.*

***Network recall**: For each class C, the fraction of data points given to an artificial neural network trained on a classification task which were truly class C, among all those which were assigned class C.*

Object semantics*: Object-type features used in machine learning models.*

Objective function*: A loss function (to be minimized during training) or its negative (to be maximized).*

Object-level training*: Training an artificial neural network to classify or predict values corresponding to objects in images or video identified by upstream processing.*

Output class*: The class which a classification model has assigned to a given input.*

Pixel-level training*: Training an artificial neural network to classify or predict values corresponding to each pixel of an image or video.*

Rectified linear units (ReLU)*: An activation function that is piecewise linear with only one non-differentiable point, at 0.*

Saliency maps*: A plot in which pixels of an image are color-coded according to the magnitude of their impact on a CNN's decision how to classify a data point.*

Softmax*: A monotone-increasing activation function which returns a value between 0 and 1 given arbitrary inputs.*

Testing set (hold-out)*: The subset of data used to test the performance (accuracy, precision, etc.) of a machine learning model on previously unobserved data once it has been trained.*

Training set*: The subset of data used to learn an optimal set of parameters in a machine learning model.*

Validation set*: The subset of data used to measure during training of a machine learning model the extent to which the model can generalize to previously unobserved data.*

***Weights**: Numerical values whose magnitude and sign determine the effect of inputs to an artificial neuron on that neuron's output.*
